# Girls referred for amenorrhea: analysis of a patient series from a specialist center

**DOI:** 10.3389/fpubh.2024.1304277

**Published:** 2024-02-16

**Authors:** Sara Mörö, Silja Kosola, Elina Holopainen

**Affiliations:** ^1^Children’s Hospital and Pediatric Research Center, University of Helsinki, Helsinki University Hospital, Helsinki, Finland; ^2^Research, Development and Innovations, Western Uusimaa Wellbeing Services County, Espoo, Finland; ^3^Department of Obstetrics and Gynecology, University of Helsinki, Helsinki University Hospital, Helsinki, Finland

**Keywords:** adolescent, amenorrhea, functional hypothalamic amenorrhea, exercise, energy deficiency, disordered eating

## Abstract

**Objective:**

Among adolescents, amenorrhea is a common reason for medical consultation. Despite the variety of underlying etiologies, the prevalence of the causes is incompletely understood. This study aimed to assess the demographic and etiological factors among patients with amenorrhea treated in a single specialist unit of adolescent gynecology.

**Design:**

Retrospective register study.

**Methods:**

Medical records of 438 girls evaluated for primary or secondary amenorrhea in a single tertiary care center between 2015 and 2019 were retrospectively reviewed. In all, 423 patients—171 with primary amenorrhea and 252 with secondary amenorrhea—were included in the study. Data on underlying conditions, anthropometric variables, and selected hormonal markers were analyzed.

**Results:**

Functional hypogonadotropic hypogonadism was the most frequent reason for primary (56%) and secondary (78%) amenorrhea. It was mostly explained by lifestyle-related functional hypothalamic amenorrhea caused by disordered eating, intense exercise, energy deficiency, psychological stress, and their combinations.

**Conclusion:**

Menstrual pattern is a significant indicator of overall health and well-being among adolescent girls and young women. Functional reasons behind primary and secondary amenorrhea are important to recognize. Treatment often requires long-term lifestyle modifications. The frequency of functional causes also implies that most amenorrhea cases are preventable.

## 1 Introduction

Menstrual disturbances are common among adolescents. Menarche is a late marker of puberty. The median age of menarche among European girls is 13.1 years (1, 2). However, about half the cycles during the first postmenarcheal years are anovulatory, and irregular menstruation may continue for several years (3). Primary amenorrhea (PA) is the absence of menarche in girls aged 15 or older with developed secondary sexual characteristics and normal growth or no signs of pubertal development in girls aged 13 or older; the latter is also classified as delayed puberty (DP) (3). Secondary amenorrhea (SA) is defined as the cessation of regular menses for a minimum of three months or the cessation of irregular menses for six months (4).

The immaturity of the hypothalamus–pituitary–gonadal (HPG) axis often explains menstrual disturbances among adolescents. However, menstrual disorders and amenorrhea can also be caused by a variety of medical conditions requiring attention or treatment. Previous studies have suggested that DP most frequently results from the constitutional delay of growth and puberty (CDGP)—an extreme variant of the normal spectrum in pubertal timing (3, 5, 6). The most common underlying causes of PA have been reported to be gonadal dysgenesis, Müllerian agenesis, and hypogonadotropic hypogonadism (3, 7, 8). However, the distribution of etiologic factors significantly varies depending on the level of previous spontaneous pubertal development. Population studies in secondary amenorrhea (SA) indicate that the most common underlying etiologies of SA after excluding pregnancy are functional disorders and polycystic ovarian syndrome (PCOS) (9–13). Although most causes of PA and SA are similar, their relative proportions may differ and are poorly described.

Although amenorrhea is a common symptom among adolescents, population-based studies on the underlying causes of PA and SA are infrequent. Etiologic diagnostics and recognition of potential lifestyle reasons contributing to menstrual problems are vital to target the treatment. We aimed to assess the demographic and etiological factors of patients with PA or SA in a cohort of girls evaluated at a single specialist care center—a direct referral center for primary care.

## 2 Materials and methods

### 2.1 Patients

This retrospective study was conducted at the Pediatric and Adolescent Gynecology Outpatient Clinic of Helsinki University Hospital.

We performed an International Classification of Diseases (ICD)-code-based inquiry (ICD codes: E30.00, E30.09, N91.0, N91.1) into the electronic patient information system and identified in all 438 girls aged 13–20 who were referred and further evaluated for delayed puberty (DP, *n* = 15), primary amenorrhea (PA, *n* = 163) or secondary amenorrhea (SA, *n* = 260) between 2015 and 2019.

PA diagnosis is used when menarche is absent either among girls aged 15 or above with developed secondary sexual characteristics and normal growth, or among girls aged 13 or above with no signs of pubertal development. SA diagnosis is used if amenorrhea has lasted over three months and the menstrual cycle was previously regular. If the menstrual cycle was previously irregular (> 40 days), SA diagnosis was set after six months of amenorrhea.

Two girls had two separate referrals during the study period and were handled in this study based on the first referral. Thirteen (3%) patients were excluded from the analysis because of missing or insufficient medical records.

In this study, all patients with DP were over 13 years old, thus fulfilling the diagnostic criteria of PA (3). Therefore, we combined DP and PA patients into one PA group (*n* = 171). In all, 252 girls presented with SA; thus, our study cohort included 423 girls.

### 2.2 Clinical data

At our center, the algorithmic approach for PA etiology described by Seppä et al. (3) is used and modified according to clinicians’ consideration. In secondary amenorrhea, a systematic approach differentiating hypo-, normo- and hypergonadotropic etiologies is used (4).

Clinical data was collected from patient records of outpatient clinic visits on the history of pubertal development, including age at Tanner stage B2 and age at menarche. In addition, data on family history of pubertal timing, past and present health, mental illness, changes in weight, eating habits, physical activity (times per week and hours per week), participation in competitive sports, and social life were collected when available. If available, the duration of amenorrhea and weight at the resumption of menses were gathered from the closest follow-up after the reported date of resumption.

The physical examination data included height and weight measurements and body mass index (BMI, kg/m^2^) calculation. BMI *z*-scores ([individual value – mean]/SD) were determined for girls up to age of 19 according to the WHO reference values based on the BMI for age and sex (14).

Documentation of the development of secondary sex characteristics of girls was made with an objective Tanner classification system (15). The gynecological examination was performed by assessing outer genitalia or, if considered clinically necessary, by pelvic examination.

Morphology of the uterus was assessed by ultrasound and compared with age and post-menarcheal values. Pelvic ultrasound examinations were performed abdominally with the conventional full bladder technique (GE Healthcare Voluson S6). Vaginal ultrasound was performed only in girls who had experienced vaginal intercourse or used tampons. The maximum anterior–posterior distance was measured in the mid-portion of the uterine body on a sagittal view, and the median length of the uterine corpus was measured from the fundus to the internal orifice of the uterus on a sagittal view. The structure of the ovaries was evaluated when technically possible.

Bone mineral density (BMD) measurement by dual-energy X-ray absorptiometry (DXA) was conducted in girls with prolonged amenorrhea (more than 12 months). BMD is reported as *z*-scores from the mean of age-, ethnicity-, and gender-related healthy individuals, and a *z*-score below – 2.0 SD is considered significantly decreased BMD (16).

Patients underwent various laboratory measurements based on clinicians’ assessments. Here, we present the clinically most relevant laboratory parameters: basal luteinizing hormone (LH), follicle-stimulating hormone (FSH), and testosterone.

The patients were classified into six main groups based on etiology (Table 1): 1. Structural, 2. Constitutional delay of growth and puberty (CDGP), 3. Permanent hypogonadotropic hypogonadism (PHH) (5), 4. Functional hypogonadotropic hypogonadism (FHH) (17), 5. Hypergonadotropic hypogonadism (Hyper H), 6. Hyperandrogenism. Since girls in our study were 13–20 years old, and ultrasound is not recommended to diagnose polycystic ovary syndrome (PCOS) in girls within eight years of menarche due to overlap with normal reproductive physiology (18), we use the term PCOS-like. We used term non-identifiable reason (NUD) in patients among whom we cannot identify an etiologic reason causing amenorrhea despite extensive examinations (groups 3, 4 and 5).

**Table 1:** The etiological classification of amenorrhea categorized into six main diagnostic groups.

1. StructuralClinicalAbsence of uterusOutflow tract problemsBiochemicalNormal FSH and LH, female-range teststerone (46 XX), male-range testosterone (46 XY)Genetic46, XX Müllerian agenesis46, XY Androgen insensitivity syndrome2. Constitutional delay of growth and puberty (CDGP)ClinicalLate puberty, spontaneous progression during follow-up and exclusion of other underlying causesBiochemicalLow FSH and LHGeneticFamily history of dealyed puberty3. Permanent hypogonadotropic hypogonadism (PHH)ClinicalPathology in the central nervous system (CNS)Organic cause without spontaneous progression of pubertal development or resolution of the menstrual cycle during follow-upNon-identifiable reason (NUD)No spontaneous recovery of menstruation during follow-up and exclusion of other underlying causesBiochemicalLow LH, FSH and estradiol levelsGeneticSingle gene mutationsSyndromes: Kallman syndrome etc.4. Functional hypogonadotropic hypogonadism (FHH)ClinicalOrganicAn identified treatable underlying organic disease, and spontaneous recovery of menstruation after the treatment of organic diseaseFunctional hypothalamic amenorrhea (FHA)Eating disorder diagnosis and exclusion of other underlying causesHistory of intense exercise and exclusion of other underlying causesHistory of weight loss and/or energy deficiency and exclusion of other underlying causesHistory of high levels of stress and exclusion of other underlying causesOverweight and exclusion of other underlying causesNon-identifiable reason (NUD)Spontaneous recovery of menstruation during follow-up and exclusion of other underlying causesBiochemicalLow or normal FSH and LHFHA
Low LH and estradiolGeneticNone5. Hypergonadotropic hypogonadism (Hyper H)ClinicalGonadal failure
BiochemicalHigh FSH, low estradiolGeneticKaryotype evaluation for Turner syndrome and Y-material6. HyperandrogenismClinicalSigns of excess androgen: acne, hirsutismOverweightPCOS-like*Gynecological ultrasound findings: *PCOS-like ovariesBiochemicalHigh testosterone, adione, 17H-progesteroneGeneticNone

FSH, follicle stimulating hormone; LH, luteinizing hormone; PCOS, polycystic ovary syndrome. *Ultrasound is not recommended to diagnose polycystic ovary syndrome (PCOS) in girls within eight years of menarche.

In the FHA group, subcategorization of lifestyle factors was based on the retrospective analysis of relevant information documented in medical records.

### 2.3 Assays

The clinical laboratory of the Helsinki University Central Hospital assayed blood samples for hormone measurements according to their standard procedures. LH levels <2 IU/L were considered to indicate hypothalamic inhibition of the HPG axis (19–21). LH/FSH relation >2 was considered suggestive of PCOS-like etiology because an elevated LH/FSH ratio is frequently observed in PCOS (22).

### 2.4 Informed consent and ethics approval

The Research Committee of the Helsinki University Hospital approved the study protocol, including the use of medical record data. Separate approval from the Ethics Committee was not needed in this register-based study, where no subjects or caregivers were contacted. All data were pseudonymized before analysis.

### 2.5 Statistical analysis

We used SPSS statistical software, release 25.0 (SPSS, Chicago, IL, USA), for statistical analyses.

Descriptive statistics included frequencies and percentages. All continuous variables were tested for normality before statistical analyses. All data are presented as medians with ranges because either data in the PA/SA or the FHA/Others groups were skewed. For comparisons of continuous variables, the Mann–Whitney U-test was used for independent samples according to distribution. The chi-squared and Fisher’s exact tests were used to analyze nominal variables. Logistic regression analysis was used to evaluate the relationship between diagnosis SA/PA and FHA/other etiologies. Stepwise logistic regression analyses were performed and the significant variables from each analysis were included in a final logistic model with FHA/other etiologies. For the stepwise selection, we used a criterion for entry of a *p-*value <0.05. The significance level was set at 0.05.

## 3 Results

Of the 423 girls, 171 (40%) had PA, and 252 (60%) had SA. The underlying causes of PA and SA were categorized into six main diagnostic groups (Table 2). The most frequent cause for PA and SA was FHH, found in 56% of PA cases and 78% of SA cases.

**Table 2:** The causes of primary (PA) and secondary amenorrhea (SA) categorized into six main diagnostic groups based on etiologic background.

	PA	SA
	*n* = 171 (%)	*n* = 252 (%)
1. Structural	11 (6)	0 (0)
Müllerian agenesis	11	
2. CDGP	43 (25)	4 (2)
3. PHH	7 (4)	2 (1)
FGFR1-mutation	2	
Septo-optic dysplasia	1	
Kallmann syndrome	1	
Previous brain surgery		1
NUD	3	1
4. FHH	95 (56)	197 (78)
Organic	4	13
FHA	90	181
NUD	1	3
5. Hyper H	5 (3)	5 (2)
POI	5	3
NUD	2	3
BPES	1	
Turner mosaicism	2	
Iatrogenic		2
History of allogenic cell transplantation		2
6. Hyperandrogenism	10 (6)	44 (17)
PCOS-like	9	41
Overweight	1	3

CDGP, constitutional delay of growth and puberty; PHH, permanent hypogonadotropic hypogonadism; FGFR1, fibroblast growth factor receptor 1; NUD, non-identifiable reason; FHH, functional hypogonadotropic hypogonadism; FHA, functional hypothalamic amenorrhea; Hyper H, hypergonadotropic hypogonadism; POI, primary ovarian insufficiency; BPES, blepharophimosis syndrome; PCOS, polycystic ovary syndrome.

Table 3 specifies the underlying conditions associated with FHH.

**Table 3:** Underlying conditions associated with FHH.

	PA	SA
	*n* = 171 (%)	*n* = 252 (%)
FHH	95 (56)	197 (78)
1. Organic	4 (2)	13 (5)
Thyroid disease		2
Hyperporlactinemia		1
DM1		2
Celiac disease	1	
IBD		2
Hydrocephalus	1	1
MCKD, dialysis	1	
NF1	1	
Disability NUD		1
Infectious disease		2
Previous use of oral contraceptives		2
2. FHA	90 (53)	181 (72)
Eating disorder	28	43
Intense exercise	15	12
ED	10	47
Psychological stress	2	10
Combination of ED and intense exercise	23	33
Combination of ED and psychological stress	2	12
Combination of intense excercise and psychological stress	1	1
Combination of intense excercise, ED and psychological stress	2	9
Overweight	4	14
3. NUD	1 (0.6)	3 (0.2)

FHH, functional hypogonadotropic; PA, primary amenorrhea; SA, secondary amenorrhea; DM, diabetes mellitus; IBD, inflammatory bowel disease; MCKD, medullary cystic kidney disease; NF, neurofibromatosis; NUD, non-identifiable reason; FHA, functional hypothalamic amenorrhea; ED, energy deficiency.

FHH was mostly explained by lifestyle-related causes categorized under FHA, accounting for 90 (53%) and 181 (72%) PA and SA cases, respectively. More specifically, amenorrhea was often caused by lifestyle factors such as intense exercise, energy deficiency (ED), and psychological stress, which explained approximately 33% of PA and 49% of SA cases. Eating disorder diagnoses were found in 28 (16%) PA and 43 (17%) SA cases. Psychological stress itself or in combination with other factors was more common among SA than PA and explicated in 4% of PA and 12% of SA cases.

Table 4 presents data pertaining to anthropometric and demographic variables, laboratory results, and medical imaging for the PA and SA groups. Girls with PA and SA entered puberty at the same age. For girls with PA who reached menarche during the study period, the median age at menarche was older than for girls with SA (17 [15–20] years vs. 13 [10–18] years, respectively, *p* < 0.001). Girls with SA developed amenorrhea on average four years (1–12 years) after menarche. During the study period, when retrospectively analyzed, the median duration of amenorrhea among 89 SA cases was found to be 20 months.

**Table 4:** Age, anthropometric variables, hormonal laboratory results, size of uterus, bone health, and lifestyle parameters in girls with PA and SA.

	PA (*n* = 171)	Data available (*n*)	SA (*n* = 252)	Data available (*n*)	*p*-value	Adjusted OR (95% CI) for SA	OR *p*-value
Age, median (range)	16 (14–21)	171	17 (12–23)	252	<0.001*	1.23^#^ (1.09–1.39)	<0.001*
Age at thelarche, median (range)	12.8 (9.5–18)	42	13 (9–14.5)	11	0.86	0.16^#^ (0.097–0.26)	<0.001*
Age at menarche, median (range)	17 (15–20)	79	13 (10–18)	225	<0.001*	0.94^#^ (0.64–1.39)	0.77
Length of amenorrhea, median months (range)			20 (4–73)	89			
Weight, median kg (range)	54.5 (35.2–123)	159	57.9 (29.8–120.3)	234	0.005*	1.021^#^ (1.007–1.036)	0.004*
Height, median cm (range)	167 (148–184)	143	167 (122–186)	207	0.36	1.01^#^ (0.98–1.04)	0.45
BMI, median kg/m^2^ (range)	19.6 (13.6–40.4)	140	20.1 (13–47.6)	203	0.03*	1.049^#^ (1.005–1.096)	0.03*
BMI <18.5, *n* (%)	38 (27)		45 (22)		0.70	0.89 (0.53–1.49)	0.66
BMI 18.5–25, *n* (%)	88 (63)		117 (58)		ref.	ref.	
BMI >25, *n* (%)	14 (10)		41 (20)		0.02*	2.2 (1.13–4.29)	0.02*
BMI, mean *z*-score for age of 5–19 years girls (SD)**	– 0.28 (1.3)	137	0.56 (1.4)	181	0.01*	1.21^#^ (1.02–1.43)	0.03*
BMI *z*-score < −2, *n* (%)	8 (5.8)		7 (3.9)		0.60	0.72 (0.25–2.05)	0.54
BMI *z*-score–2–1, *n* (%)	111 (81)		135 (74.6)		ref.	ref.	
BMI *z*-score > 1, *n* (%)	18 (13.1)		39 (21.5)		0.07	1.78 (0.97–3.29)	0.07
Hormone measurements, median (range)
FSH, IU/L	5.9 (0.02–78.7)	246	5.4 (0.1–145)	209	0.1	0.99^#^ (0.97–1.01)	0.56
LH, IU/L	4.5 (0.006–66.6)	246	3.4 (0.02–40.3)	211	0.09	0.98^#^ (0.95–1.02)	0.31
LH < 2 IU/L, *n* (%)	30 (21)		70 (33)		0.01*	1.96 (1.16–3.33)	0.01*
Testosterone, nmol/L	1 (0.4–5.3)	19	1.5 (0.2–8.2)	39	0.02*	1.71^#^ (0.88–3.33)	0.12
Uterus measurements, median (range)
AP of corpus of uterus, mm	21 (6–40)	153	23 (14–44)	223	0.001*	1.05^#^ (1.01**–**1.09)	0.01*
Length of corpus of uterus, mm	39 (20–62)	208	40 (26–67)	227	0.17	1.024^#^ (0.99–1.06)	0.19
Thickness of endometrium, mm	4 (0.8–16)	124	4 (0.9–18.3)	196	0.97	0.995^#^ (0.903–1.097)	0.93
BMD L1–4, median *z*-score (range)	−1.1 (−3.1–1.4)	52	−1.2 (−5.3–1.9)	67	0.71	1.086^#^ (0.79–1.50)	0.61
BMD L1–4, *z*-score < −2, *n* (%)	13 (25)		15 (22)		0.83	1.16 (0.49–2.71)	0.74
BMD TBLH, median *z*-score (range)	−0.2 (−3.1–1.4)	29	0.2 (−5–3)	36	0.43	1.17^#^ (0.79–1.72)	0.44
BMD TBLH, *z*-score < −2, *n* (%)	3 (10)		2 (6)		0.65	1.96 (0.31–12.6)	0.48
Stress fractures, *n* (%)	10 (44)	23	9 (30)	30	0.39	1.80 (0.58–5.59)	0.31
Regular physical activity outside school, median times/week (range)	4 (1–11)	98	4 (1–10)	135	0.21	0.93^#^ (0.81–1.06)	0.28
Regular physical activity outside school, median hours/week (range)	12 (2–31)	38	7 (2–35)	38	0.01*	0.94^#^ (0.89–1.00)	0.05*
Competitive sport, *n* (%)	34 (100)	34	26 (70)	37	<0.001*		

PA, primary amenorrhea; SA, secondary amenorrhea; OR, odds ratio; CI, confidence interval; BMI, body mass index; SD, Standard deviation; FSH, follicle-stimulating hormone; LH, luteinizing hormone; AP, anteroposterior diameter; BMD, bone mineral density; TBLH, total body less head. ^#^OR for unit to increase in continuous variables. *Statistically significant *p-*value. ** > 19 years old girls in PA/SA groups = 10/30.

Median weight, BMI and BMI *z*-score values were lower among girls with PA than with SA; in turn, BMI over 25 kg/m^2^ was more common among girls with SA than PA (OR 2.2, 95% CI 1.13–4.29, *p* = 0.02). Still, most of the amenorrheic girls were normal weight (BMI 18.5–25 kg/m^2^, *z*-score − 2–1). A weight increase of 2.3 kg (0–10) was reported at the commencement of the menses in seven PA girls. Among 39 girls with SA, variation in weight change reported before recovery of the menstrual cycle was wide (range −17 – +23 kg).

We found no statistical difference in basal LH and FSH measurements between PA or SA girls. However, LH levels <2 IU/L were more common in the SA (33%) group than in the PA group (21%) (OR 1.96, 95% CI 1.16–3.33, *p* = 0.01). No significant difference was found in LH/FSH ratios or LH/FSH > 2 between the amenorrheic groups. Testosterone levels among the SA girls were significantly higher than among PA girls (*p* = 0.02). However, median values were in the normal range in both groups.

Girls with PA had a slightly smaller uterus than girls with SA. No significant differences in bone health were found between the PA and SA groups.

As Table 4 shows, PA girls reported spending more time in regular physical activities and more frequent participation in competitive sports than SA girls (*p* = 0.01 and *p* < 0.001, respectively).

We also analyzed a whole group of girls with FHA (*n* = 271), including those who presented with PA and those with SA, and compared it to girls with other etiologies (*n* = 152) of amenorrhea (Table 5). FHA girls were significantly leaner than girls with other etiologies of amenorrhea. FHA girls also had low basal LH levels more often than girls with other etiologies (37% vs. 11%). Although we found no statistically significant difference in the number of stress fractures among amenorrheic girls, 16 out of 19 stress fractures were reported in the FHA group. Girls with FHA more often reported participation in competitive sports and had significantly more physical activity (times per week) than girls with amenorrhea of other etiology (OR 1.36, 95% CI 1.14–1.61, *p* < 0.001).

**Table 5:** Clinical data and lifestyle parameters among girls with FHA compared to girls with amenorrhea of other etiology.

	FHA (*n* = 271)	Data available (*n*)	Others (*n* = 152)	Data available (*n*)	*p-*value	Adjusted OR (95% CI) for FHA	OR *p*-value
Age, median (range)	17 (13–23)	271	17 (12–20)	152	0.37	1.07^#^ (0.95–1.21)	0.29
BMI, median kg/m^2^ (range)	19.6 (13–47.6)	228	20.7 (15.9–40.5)	115	<0.001*	0.95^#^ (0.91–0.99)	0.006*
BMI <18.5, *n* (%)	64 (28)		19 (17)		<0.001*	1.60 (0.89–2.89)	0.12
BMI 18.5–25, *n* (%)	139 (61)		66 (57)		ref.	ref.	
BMI >25, *n* (%)	25 (11)		30 (26)		<0.001*	0.40 (0.22–0.73)	0.003*
BMI, median *z*-score for age of 5–19 years (range)**	−0.47 (−3.9–4.8)	209	−0.5 (−2.2–3.2)	109	<0.001*	0.74^#^ (0.62–0.87)	<0.001*
BMI *z*-score < −2, *n* (%)	12 (6)		3 (3)		0.56	1.72 (0.47–6.28)	0.41
BMI *z*-score − 2–1, *n* (%)	172 (82)		74 (68)		ref.	ref.	
BMI *z*-score > 1, *n* (%)	25 (12)		32 (29)		<0.001*	0.34 (0.19–0.61)	<0.001*
LH < 2 IU/L, *n* (%)	86 (37)	230	13 (11)	127	<0.001*	5.35^#^ (2.77–10.35)	<0.001*
Uterus measurements, median (range)							
AP of corpus of uterus, mm	22 (6–44)	249	23 (9.5–40)	127	0.039*	0.98^#^ (0.95–1.01)	0.25
Length of corpus of uterus, mm	39 (20–67)	143	40 (24–58)	82	0.59	0.99^#^ (0.96–1.03)	0.73
Thickness of endometrium, mm	4 (0.9–18.3)	204	4.6 (0.8–11.9)	113	0.29	0.97^#^ (0.77–1.07)	0.54
BMD L1-4, median *z*-score (range)	−1.3 (−4.2–1.5)	83	−1 (−5.3–1.9)	36	0.14	0.81^#^ (0.56–1.15)	0.24
BMD TBLH, median *z*-score (range)	−0.2 (−3.1–3)	46	0.3 (−5–1.4)	19	0.67	1.06^#^ (0.70–1.60)	0.80
Stress fractures, *n* (%)	16 (35)	46	3 (43)	7	0.65	0.71 (0.14–3.58)	0.68
Regular physical activity outside school, times/week (range)	4 (1–11)	163	3 (1–8)	70	<0.001*	1.36^#^ (1.14–1.61)	<0.001*
Regular physical activity outside school, hours/week (range)	11.3 (2–35)	59	7 (2–24)	17	0.07	1.08^#^ (0.995–1.17)	0.07
Competitive sport, *n* (%)	52 (84)	62	8 (13)	9	1.00	0.70 (0.17–13.70)	0.70

FHA, Functional hypothalamic amenorrhea; OR, Odds Ratio; Cl, confidence interval; BMI, body mass index; SD, Standard Deviation; LH, luteinizing hormone; AP, anteroposterior diameter; BMD, bone mineral density; TBLH, total body less head. ^#^OR for unit to increase in continuous variables. *Statistically significant *p-*value. ** > 19 years old girls in Others/FHA groups = 12/28.

In stepwise logistic regression, LH < 2 IU/L was the strongest predictor for diagnosis, with an odds ratio of 5.64 (CI 95% 2.12–15.05, *p* < 0.001) for FHA. Also, frequent physical activity was associated with FHA (OR 1.48, CI 95% 1.19–1.84; *p* < 0.001 for FHA). BMI categories (either kg/m^2^ or *z*-scores) showed no significant association with FHA diagnosis (Table 6).

**Table 6:** Binary logistic regression analysis of factors influencing functional hypothalamic amenorrhea (FHA) in comparison to other etiologies.

Independent variable	Adjusted OR (95% CI)	*p-*value
BMI, kg/m^2^		0.14
<18.5 kg/m^2^	2.61 (0.97–6.99)	0.057
18.5–25 kg/m^2^	ref.	
>25 kg/m^2^	0.71 (0.12–4.37)	0.71
LH < 2 IU/L (<2 versus ≥2)	5.64 (2.12–15.05)	<0.001*
Regular physical activity outside school, times/week	1.48^#^ (1.19–1.84)	<0.001*

OR, odds ratio; CI, confidence interval; BMI, body mass index; LH, luteinizing hormone. ^#^OR for unit to increase in continuous variables. *Statistically significant *p*-value.

## 4 Discussion

In this study, the evaluation of amenorrhea among adolescent girls revealed that FHH, particularly its sub-category FHA, was the most frequent cause among girls with PA and SA. In both groups, lifestyle-related reasons such as intense exercise, energy deficiency, and/or psychological stress were notably high, while organic reasons were rare. Intense exercise alone or combined with other lifestyle-related reasons (24%) and participation in competitive sports were especially common among girls with PA.

Adolescence is a vulnerable time for sensitive HPG axis, affecting pubertal development, timing of menarche, and development of regular menstrual cycle. Adolescent morbidity has risen; its leading cause is mental health problems (23). This trend underscores the increasing challenges and stressors adolescents encounter and their struggles in managing them in today’s world. In our study, FHA stands out as a prominent etiological factor, particularly in cases of secondary amenorrhea, aligning with the prevailing clinical and research knowledge of amenorrhea (9, 11). FHA is often associated with stressors like disordered eating, weight loss, excessive exercise, and plain stress caused by common life events (9). Women with FHA exhibit more dysfunctional attitudes, such as perfectionism, a high need for social approval, and lofty expectations for self and others (24); they also cope less well with stress (25). Prior studies suggest the contribution of genetics controlling the development and/or function of GnRH neurons, particularly to FHA, and, therefore, explain part of the personal predisposition to stress-related FHA (26). In this study, a negative energy balance was found to be a common reason causing amenorrhea, and amenorrheic girls were particularly lean. Eating disorders behind amenorrhea were less frequent in our study than in previous studies, where different eating disorders were diagnosed behind 68% of SA (12). However, 17% of girls in our study population had an eating disorder compared to 6% in the general Finnish population of 20–35-year-old women (27).

Lifestyle-related reasons for amenorrhea among adolescents may increase with age. In a study from Thailand, the most common etiology behind PA was Mullerian agenesis (39.7%), followed by gonadal dysgenesis (35.3%) (8). Only in 0.3% of cases was hypothalamic dysfunction reported (8), indicating the importance of genetic diversity and cultural and environmental factors in the etiology of PA. In a large patient series collected in the 1980s, the four most common causes of PA were ovarian failure (48.5%), congenital absence of uterus (16.2%), GnRH deficiency (8.3%), and CDGP (6%) (28), meaning the proportion of functional reasons behind amenorrhea has increased over time, or they are better recognized. However, categorizing amenorrhea is complex due to the partial overlap of underlying causes and multiple contributing factors. Our findings of a high prevalence of FHA and PCOS-like situations behind SA are consistent with earlier observations (12, 13). In this study, some secondary amenorrhea patients were classified under the CDGP group based on the treating gynecologists’ interpretation of irregular menses as a normal irregularity linked to the late onset of menstruation.

In this study, the onset of puberty settled at the upper limit of the population’s normal range and SA girls’ menarche at the population average (3). Sedlmeyer et al. and Varimo et al. showed in their large patient series from the Pediatric Endocrine Clinics that CDGP (30 and 56%, respectively) was the most common etiology behind delayed puberty in females; FHH was found in only 19 and 20% of girls with delayed puberty (5, 6). In our study from the adolescent gynecology outpatient clinic, the proportion of FHH in the PA group was markedly higher than CDGP, and lifestyle-related reasons predominated.

Early-onset estrogen deficiency is associated with compromised uterine growth and could affect subsequent reproductive function (29, 30). This study found no statistically significant differences in the adverse effects of hypoestrogenism in uterus sizes among amenorrheic girls. However, the uterus sizes of amenorrheic girls in previous studies were significantly smaller than in girls of the same chronological and gynecological age with regular menstrual cycles (29–31). Although no significant differences in BDM levels between PA and SA girls were found, amenorrhea and low estrogen levels negatively affect bone health, especially during puberty. Moreover, despite weight and menstrual recovery, individuals who experience bone loss as adolescents have chronic deficits and an increased risk of fractures in adulthood (32).

Most data concerning the etiologic factors of amenorrhea are based on clinical practice and review articles handling also evaluation and management of amenorrhea (3, 9, 11, 19). We believe extensive patient series-based evaluations are sparse (5, 6, 8, 12, 13). Our study’s strength lies in its patient series approach, featuring a nonselective patient cohort from a public referral center for the metropolitan Helsinki area (background population of 1.4 million). Comparing our findings with a previous patient series shows that the distribution of the prevalence of etiological reasons, especially behind primary amenorrhea, varies depending on the study population (5, 6, 8, 28). In Finland, PA patients with growth delay and/or lack of any marks of spontaneous pubertal development are often initially referred to a pediatric endocrinologist (6), and patients with pubertal development spontaneously starting are more often examined and treated by a gynecologist, which may explain the differences. This study’s limitation mainly stems from its retrospective design, explaining the amount of missing data. Clinical data were based on a review of electronic patient records, and no validated questionnaires were systematically used to diagnose eating disorders, dietary habits, psychological stress or physical exercise levels. Variations in medical record practices lead to a limited availability of data; therefore, our results must be interpreted with caution.

In conclusion, lifestyle habits, including disordered eating, stress, weight loss, and excessive exercise, seem particularly important to identify among adolescent girls. Early recognition and timely management by a multidisciplinary team are crucial to prevent the severe and long-term effects of these conditions. Our study has major implications for public health because the frequency of functional causes implies that most amenorrhea cases are preventable.

## Data availability statement

The datasets presented in this article are not readily available because restrictions apply to the availability of data generated or analyzed during this study to preserve patient confidentiality. The corresponding author will on request detail the restrictions and any conditions under which access to some data may be provided. Requests to access the datasets should be directed to elina.holopainen@hus.fi.

## Ethics statement

Ethical approval was not required for the studies involving humans because the Research Committee of the Helsinki University Hospital approved the study protocol, including the use of medical record data. Separate approval from the Ethics Committee was not needed in this register-based study, where no subjects or caregivers were contacted. All data were pseudonymized before analysis. The studies were conducted in accordance with the local legislation and institutional requirements. Written informed consent for participation was not required from the participants or the participants’ legal guardians/next of kin in accordance with the national legislation and institutional requirements because separate informed consent was not needed in this register-based study, where no subjects or caregivers were contacted. All data were pseudonymized before analysis.

## Author contributions

SM: Conceptualization of study, Formal analysis, Writing – original draft, Writing – review & editing. SK: Conceptualization of study, Supervision, Writing – original draft, Writing – review & editing. EH: Conceptualization of study, Supervision, Writing – original draft, Writing – review & editing.

## Glossary

**Table:** 

PA	Primary amenorrhea
DP	Delayed puberty
SA	Secondary amenorrhea
HPG axis	Hypothalamus-pituitary-gonadal axis
CDGP	Constitutional delay of growth and puberty
PCOS	Polycystic ovary syndrome
ICD	International Classification of Diseases
BMI	Body mass index
BMD	Bone mineral density
DXA	Dual-energy X-ray absorptiometry
LH	Luteinizing hormone
FSH	Follicle-stimulating hormone
CNS	Central nervous system
PHH	Permanent hypogonadotropic hypogonadism
FHH	Functional hypogonadotropic hypogonadism
FHA	Functional hypothalamic amenorrhea
NUD	Non-identifiable reason
Hyper H	Hypergonadotropic hypogonadism
SD	Standard deviation
ED	Energy deficiency
